# Caffeic Acid Phenethyl Ester as a Protective Agent against Nephrotoxicity and/or Oxidative Kidney Damage: A Detailed Systematic Review

**DOI:** 10.1155/2014/561971

**Published:** 2014-06-03

**Authors:** Sumeyya Akyol, Veli Ugurcu, Aynur Altuntas, Rukiye Hasgul, Ozlem Cakmak, Omer Akyol

**Affiliations:** ^1^Division of Medical Laboratory Techniques, Vocational School of Health Sciences, Turgut Ozal University, Çeşme Durağı Spor Kompleksi Arkası, Ostim, Yenimahalle, 06370 Ankara, Turkey; ^2^Department of Biochemistry, Medical Faculty, Gaziosmanpasa University, Tokat, Turkey; ^3^Private Bilecik Orhangazi Dialysis Center, Bilecik, Turkey; ^4^Ankara Branch of Council of Forensic Medicine, Ankara, Turkey; ^5^Department of Biology, Faculty of Education, Gazi University, Ankara, Turkey; ^6^Department of Medical Biochemistry, Medical Faculty, Hacettepe University, Ankara, Turkey; ^7^Division of Chemistry, Ankara Branch of Council of Forensic Medicine, Ankara, Turkey

## Abstract

Caffeic acid phenethyl ester (CAPE), an active component of propolis, has been attracting the attention of different medical and pharmaceutical disciplines in recent years because of its antioxidant, anti-inflammatory, antiproliferative, cytotoxic, antiviral, antifungal, and antineoplastic properties. One of the most studied organs for the effects of CAPE is the kidney, particularly in the capacity of this ester to decrease the nephrotoxicity induced by several drugs and the oxidative injury after ischemia/reperfusion (I/R). In this review, we summarized and critically evaluated the current knowledge regarding the protective effect of CAPE in nephrotoxicity induced by several special medicines such as cisplatin, doxorubicin, cyclosporine, gentamycin, methotrexate, and other causes leading to oxidative renal injury, namely, I/R models and senility.

## 1. Introduction

Epidemiological data show that mortality resulting from kidney diseases has been growing in recent years, although other causes of mortality such as cerebrovascular, heart, and infectious diseases are significantly decreased. Thus, the investigations of new biologically active compounds are very important for the development of novel medications to treat kidney diseases.

**Figure 1:**
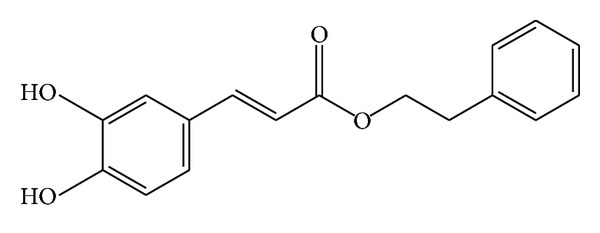
The chemical structure of caffeic acid phenethyl ester (CAPE).

**Figure 2:**
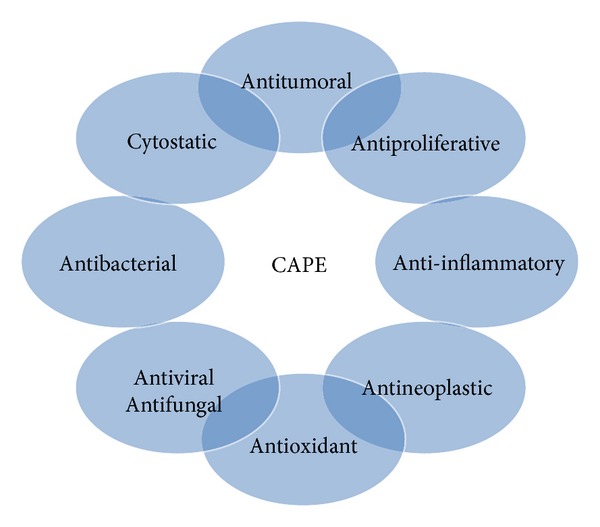
The biological effects of caffeic acid phenethyl ester (CAPE)* in vivo* and* in vitro*.

Caffeic acid phenethyl ester (CAPE) has been a widely used folk medicine in Asia for thousand years. It is an active phenolic part of propolis (Figure 1) in the hives of honeybees and possesses important physiological activities, including antitumoral, antiproliferative, anti-inflammatory, antineoplastic, and antioxidant properties (Figure 2). The composition of propolis has a common structure containing waxes, resin, and volatiles but the whole content is complex and varies according to the geographical area. The ingredients in propolis resin include phenolic acids, their esters, terpenes, flavonoids, fatty acids, steroids, aromatic aldehydes, and alcohols. CAPE is the most studied substance among these components of propolis. The molecular weight and empirical formula of CAPE are 284.3 g/mol and C_17_H_16_O_4_. All flavonoids within propolis, but not CAPE, are reported to have a low order of acute oral toxicity with a reported LD50 of 8–40 g/kg [1]. Similarly, a safe dose in humans is estimated as 1.4 mg/kg body weight/day, or approximately 70 mg/day [1].

CAPE has antioxidant characteristics via inhibition of reactive oxygen species (ROS) production in human neutrophil cells and in the xanthine oxidase (XO) systems at a concentration of 10 *μ*M [2]. It is a potent inhibitor of NF-*κ*B [3, 4]. In the last two decades, a growing number of* in vivo* studies have focused on the protective effect of CAPE on several disease models in rats, mice, and rabbits (Table 1). Lately, there have been several publications focusing on the protective effect of CAPE in kidney pathologies and nephrotoxicity [5–8]. The* in vivo* nephroprotective effects of CAPE in several kidney pathologies are summarized with details such as doses applied, type of action, and reported outcomes in Table 2. Several proposed molecular protection mechanisms of CAPE have been suggested by* in vivo* and* in vitro* studies. A general evaluation of the nephroprotective activities of CAPE has not been published before. Therefore, in this paper, we discuss the potential nephroprotective action of CAPE and its molecular mechanisms in a variety of kidney pathologies.

*The Cytotoxic Effect of CAPE.* Besides antioxidant, anti-inflammatory, antiproliferative, and other preventive effects, CAPE has also cytotoxic effects in some specific situations. Grunberger et al., one of the leading groups working on CAPE, did the first cytotoxicity study on normal rat/human versus transformed rat/human melanoma and breast carcinoma cell lines [9]. In addition to direct cytotoxic effects on cancer cells, CAPE also sensitizes cancer cells to the chemotherapeutic drugs and radiation by inhibiting biochemical pathways that lead to treatment resistance [10]. As a dramatic example, CAPE was found to enhance the antiproliferative and cytotoxic effects of docetaxel and paclitaxel in prostate cancer cells [11]. This was attributed to CAPE augmentation of docetaxel and paclitaxel proapoptotic effects in addition to the CAPE-induced increase of estrogen receptor alpha and beta levels. To study the mechanism of differential cytotoxicity of CAPE, researchers used normal CREF cells and adenovirus-transformed CREF cells (Wt3A) [12]. They studied whether CAPE-induced toxicity was influenced by the redox state of the cell (e.g., depletion of GSH and hydrogen peroxide (H_2_O_2_) application to the cells were analyzed) to explore possible mechanisms of CAPE-induced apoptosis. They found that CAPE can modulate the redox state of cells and the sensitivity of cells to CAPE-induced cell death may be determined by the loss of normal redox state regulation of transformed cells. CAPE and its ethyl analogue have been shown to have significant cytotoxicity on oral submucosus fibroblast, gingiva carcinoma, and tongue squamous cell carcinoma cells [13]. Apoptosis induced by CAPE was found to be associated with mitochondrial dysfunction, GSH depletion, and selective scavenging of hydrogen peroxide in human leukemic HL-60 cells [14]. Growth of a human glioblastoma multiforme cell line was suppressed by CAPE in a dose-dependent manner [15]. Tumor suppressor proteins p53 and p38 MAPK play prominent roles in CAPE-induced apoptotic cell death that might contribute to the antineoplastic effects of CAPE [16]. Lately, it was found that CAPE has time- and dose-dependent cytotoxic effects on CCRF-CEM leukemic cells [17]. The major molecular fundamentals of cytotoxic effects of CAPE in terms of protein kinases, antiapoptotic and apoptotic proteins, growth factor pathways, transcription factors, cell cycle proteins, cell adhesion molecules, metastasis, and some others have extensively been reviewed by Akyol et al. [18].

**Figure 3:**
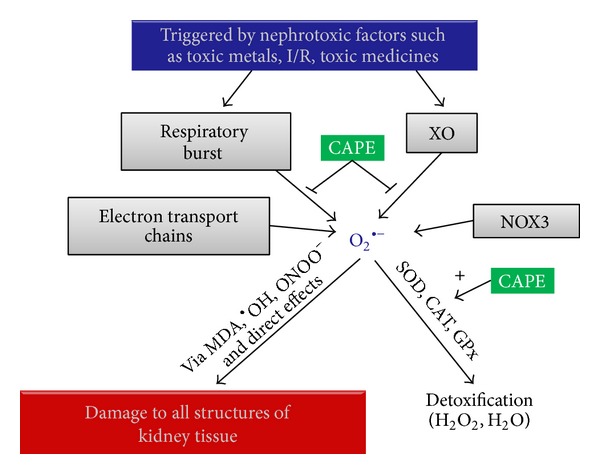
Proposed mechanism for the relationship between oxidative stress and the protective effect of CAPE on kidney. Abbreviations: CAPE: caffeic acid phenethyl ester, CAT: catalase, GPx: glutathione peroxidase, H_2_O: water, H_2_O_2_: hydrogen peroxide, I/R: ischemia/reperfusion, MDA: malondialdehyde, NOX3: NADPH oxidase 3, O_2_^∙−^: superoxide anion radical,^∙^OH: hydroxyl radical, ONOO^−^: peroxynitrite, SOD: superoxide dismutase, XO: xanthine oxidase.

*The Mechanisms of Renal Protection Afforded by CAPE.* There is an increasing body of evidence that XO has an important role as the primary source of ROS in the ischemic kidney (Figure 3). Oxidative stress from other sources such as toxic medicine and toxic metals can result from increased ROS production and/or from decreased ROS scavenging capability. In ischemic conditions, ATP is degraded to hypoxanthine, and correspondingly xanthine dehydrogenase is converted to XO [19]. During reperfusion, XO catalyzes the conversion of hypoxanthine to uric acid with release of the superoxide radical (O_2_^∙−^). Subsequent reactions catalyzed by superoxide dismutase (SOD) result in the formation of H_2_O_2_, which is less hazardous than O_2_^∙−^. Hydrogen peroxide is then converted to H_2_O and molecular oxygen (O_2_) by CAT (Figure 3). It is also known that neutrophil infiltration was stimulated in the ischemic kidney and after damages by other factors than ischemia. Such an infiltration might be regarded as another source of free radicals in the kidney because activation of neutrophils results in the production and release of potentially toxic oxygen metabolites, especially O_2_^∙−^ (Figure 3) [19]. The integrity of nephron cells is supplied by the cell membrane. Normal functions of the cells are maintained when lipid and protein structures of the membrane are intact. The peroxidation of unsaturated fatty acids with ROS occurs as a chain reaction, and, if severe, it destroys membrane integrity (Figure 3). MDA is particularly released as a result of toxic effects of active oxygen radicals, which destroy unsaturated fatty acids in the cell membrane. On the other hand, O_2_^∙−^ have been reported to react with NO to produce peroxynitrite (ONOO^−^) that can decompose to form nitrogen dioxide (NO_2_) and hydroxyl radical (^∙^OH). Peroxynitrite and its further products have been linked to several interactions that may contribute to cellular injury, including lipid peroxidation, nitrosylation of some molecules, and interactions with different metals that have redox potential, such as iron and copper [20]. ONOO^−^ has been hypothesized to play an important role in renal I/R injury [21]. ONOO^−^ generated in the tubular epithelium during I/R or cisplatin administration has the potential to impair the adhesion properties of tubular cells, which then may contribute to the tubular obstruction in acute renal failure [22].

CAPE exhibits antioxidant properties by blocking production of ROS in human neutrophils and the xanthine-xanthine oxidase system [2] (Figure 3). CAPE has a higher hydrophobicity and stronger inhibition potency toward XO and it inhibits the enzymatic activity via binding to the molybdopterin region of its active site. We have previously reported in I/R models of several organs that CAPE caused an elevation in CAT [23, 24] and SOD activities [24]. It is not known yet whether CAPE has a regulatory effect on antioxidant enzyme activity. However, it has been speculated that CAPE may affect the transcriptional and/or translational pathways of these antioxidant enzymes (Figure 3). Because CAPE has anti-inflammatory, immunomodulatory, antiproliferative, and antioxidant properties and has been shown to inhibit lipoxygenase activities, as well as suppress lipid peroxidation [2, 23, 24], it can easily suppress lipid peroxidation in renal tissue.

## 2. Protective Efficacy of CAPE in Kidney Pathologies

### 2.1. Ischemia/Reperfusion (I/R) Injury in the Kidney

I/R injury in the kidney is one of the complications of renal operations. As a second phase after ischemia, ROS and activated neutrophils increase the damage of early ischemic injury in the reperfusion state. During ischemia to any organ, including the kidney, ATP is degraded to ADP and eventually to AMP. It is then converted to adenosine after removal of the phosphate group by hydrolytic cleavage. Then, adenosine is deaminated into inosine by an enzyme, adenosine deaminase (ADA). After this step, inosine is converted to hypoxanthine and finally it is turned to xanthine and uric acid by XO. The last enzyme is a major source of ROS, especially superoxide radicals [25, 26]. We conducted a study to investigate whether pretreatment of animals with CAPE modifies the level of endogenous indices of oxidant stress markers like XO, malondialdehyde (MDA), nitric oxide (NO), and ADA [5]. In this study and others, we used a 10 *μ*mol/kg i.p. dose of CAPE, which is equal to approximately 2.85 mg/kg. CAPE was administered to the animals in the dose of 30 mg/kg/day (approximately 0.1 mmol/kg/day), orally by some authors [27]. We found that a renal artery occlusion for 30 minutes did not significantly change the levels of the parameters studied in serum except NO. However, subsequent reperfusion led to significantly elevated MDA levels and reduced XO activity. Pretreatment of rats with CAPE reversed these changes. CAPE also led to a significant increase in NO levels compared to sham and reperfusion groups. Reperfusion triggered the declining of ADA activity in serum but CAPE did not change the level keeping the activity lower than sham and ischemia groups. As an important point, a marked decrease in XO activity means that O_2_^∙−^ stem from neutrophils rather than from the XO system in kidneys. We suggested that the neutrophil-based generation of O_2_^∙−^ is the major source of reperfusion injury in the kidney and that the cytoprotective effect of CAPE may also stem from the interaction with neutrophils attenuating neutrophil-mediated injury. Renal ischemia induced NO production but reperfusion did not change the levels of this enzyme found in ischemia. In turn, CAPE led to an increase in the NO levels compared to I/R groups. However, NO consumption by ROS in the kidney is prevented by CAPE action or CAPE stimulated the release of NO from the endothelial cells. Another investigation focused on the protective effect of CAPE on ipsilateral and contralateral rat kidneys via NO and myeloperoxidase (MPO) pathways [6]. There were statistically significant increases in tissue NO concentrations of ischemia, I/R, and CAPE groups in the contralateral kidney but not in the ipsilateral kidney. MPO activity decreased after ischemia in the ipsilateral kidney and, upon reperfusion, it increased. After treating animals with CAPE, the process was attenuated serving as a protective element to protect kidney tissue from damage. Ipsilateral kidney was seen to have elevated MPO activities compared to the contralateral kidney. SOD activities were increased in I/R and CAPE groups with respect to sham operation group in ipsilateral kidneys. When ipsilateral and contralateral kidneys were compared, SOD activity was found to be higher in I/R and CAPE groups in the ipsilateral kidney. We concluded that CAPE would be helpful in clinical practice, for example, at reconstructive renal surgery and transplantation. In another study, we aimed to determine whether CAPE offers any advantage over *α*-tocopherol on an* in vivo* model of I/R injury in rats [19]. CAPE at 10 *μ*mol/kg or *α*-tocopherol at 10 mg/kg was administered i.p. before the reperfusion. Acute administration of CAPE was found to suppress I/R-induced renal lipid peroxidation and tissue injury more effectively than *α*-tocopherol.

Lately, a study was designed in rats to investigate the effect of single i.p. injection of 10 *μ*mol/kg CAPE under isoflurane anesthesia on the kidney [28]. The right kidney was removed from all animals. Injections were administered 40 min before left renal ischemia. At the end of the experiments, carotid artery blood was taken for creatinine analysis and kidney was removed for histological analysis of hematoxylin and eosin-stains. Creatinine values in the CAPE group were higher at the end of the procedure. Histopathological examination showed the CAPE group to display more pericapsular tubular necrosis compared to the control and ethanol-given groups. The researchers concluded that CAPE promoted greater functional and anatomic renal injury when rats were anesthetized with isoflurane, hypothesizing that CAPE scavenged ROS and blocked the anesthetic preconditioning by isoflurane.

During the myocardial I/R, peripheral blood flow is reduced in a time coursing manner from ischemia to reperfusion. This may cause damage in the kidney as well because of being the most perfused organ in the body. In normal functioning kidneys, the ROS generated by kidney are well metabolized by the adaptive scavenger mechanism but excessive ROS may cause acute or progressive renal damage in endogenous or exogenous renal injury [29]. This damage may arise from hypoperfusion, hemolysis, and systemic inflammatory answer during myocardial revascularization and disappearance of pulsatile perfusion [30]. A study by Ozer et al. indicated that myocardial I/R injury significantly increased MDA and decreased the glutathione (GSH) content of kidney [31]. They applied CAPE intravenously at a 50 *μ*mol/kg concentration starting 10 min before occlusion and ending 30 minutes after ischemia. Histopathological examination of kidneys has also shown that myocardial I/R caused severe renal damage. CAPE significantly decreased MDA production and increased GSH content as well as moderated morphological damage originated from myocardial I/R in kidneys.

### 2.2. Cyclosporine Nephrotoxicity

Cyclosporine A (CsA), a potent immunosuppressive drug, was initially isolated from the fungus* Tolypocladium inflatum* and has been used to treat autoimmune diseases and to prevent rejection of organ transplants. Treatment with CsA may be associated with a number of potentially serious adverse effects. It is nephrotoxic and neurotoxic and causes hypertension because of renal vasoconstriction, and it may lead to squamous cell carcinoma and infections. The serious nephrotoxic effects of CsA limit its use and therapeutic benefits [32]. The hypothesis that CAPE is a potential protective agent against CsA nephrotoxicity due to its antioxidant properties was examined by evaluating the renal function, morphology, and oxidative stress status using a rat model of CsA-induced nephrotoxicity [33]. It was found that CsA-treated rats decreased food intake and lost weight whereas these disturbances were significantly improved by concomitant treatment with CAPE regardless of the CAPE concentration. Blood urea nitrogen (BUN) and serum creatinine levels increased upon CsA administration but this elevation was reversed by CAPE pretreatment. On the other hand, CsA led to reductions in urea and creatinine clearances while an increase in urine flow rate and fractional sodium excretion was observed. All these changes were reversed by CAPE pretreatment. There was a significant increase of renal MDA and decrease of renal GSH levels in CsA-treated rats, whereas CAPE application prevented the changes in these parameters. The rat kidney treated with CsA demonstrated diffuse transcortical vacuolar degeneration and focal necrosis of proximal tubular cells with focal dystrophic calcification. Most of the nuclei of the proximal tubular cells showed degeneration including pyknosis, karyorrhexis, and karyolysis. These structural changes were markedly prevented by CAPE pretreatment. All the above-mentioned findings suggest that CAPE may be a promising agent for attenuation of the side effects of CsA on kidney at least in part by inhibiting renal lipid peroxidation and enhancing or maintaining the antioxidant GSH content. In another study [34], the effect of CAPE was investigated in CsA-induced nephrotoxicity in rats. CsA administration caused renal damage, which was quantified by a significant increase in serum BUN and creatinine levels. CAPE treatment prevented the increase in creatinine levels but did not improve the renal function efficiently. It was demonstrated that MPO, SOD, and CAT activities increased in rat renal tissue under CsA treatment. CAPE attenuated these increases, resulting in renal protection against CsA toxicity.

### 2.3. Cisplatin, Doxorubicin, and Methotrexate Nephrotoxicity

Cisplatinum or cis-diamminedichloroplatinum(II) (CDDP) is a member of a class of platinum-containing anticancer drugs causing crosslinking of DNA and triggering apoptosis. It is used to treat various types of cancers including carcinomas (ovarian cancer and small cell lung cancer), lymphomas, germ cell tumors, and sarcomas. CDDP has a number of side effects that can limit its use. The most known side effects are nephrotoxicity, neurotoxicity, nausea and vomiting, ototoxicity, electrolyte disturbances, myelotoxicity, and hemolytic anemia. Nephrotoxicity is the most common and important adverse effect and it is a major concern. The mechanism of nephrotoxicity seems to be related to ROS. If creatinine clearance is reduced, the dose should be reduced to protect the kidney. There have been a number of attempts to prevent this adverse effect by using free radical scavenging agents like CAPE. We designed a study to investigate whether treatment of rats with CAPE prior to CDDP administration prevents CDDP-induced nephrotoxicity [7]. For this purpose, we have investigated the histopathological effects of CDDP and the possible protective effect of CAPE on renal damage. We also examined various markers for oxidative stress status such as SOD, CAT, GSH-Px, MDA, NO, and MPO in rat renal tissues subjected to CDDP-induced toxicity with or without pretreatment with CAPE. CDDP administration at a dose of 7 mg/kg body weight resulted in acute renal failure. Plasma BUN and creatinine levels were increased after CDDP application. CAPE attenuated the increase in serum BUN level. NO and MDA levels in kidney tissue were increased by CDDP administration; again, CAPE prevented these elevations. In the CDDP group of rats, antioxidant enzyme activities (SOD, CAT, and GSH-Px) were decreased, but in the CAPE group these enzymes were found to be unchanged. In histopathological analysis, CDDP administration to the rats formed a remarkable proximal tubular necrosis with extensive epithelial vacuolization, swelling, and tubular dilatation compared to CAPE plus CDDP-treated rats. We concluded in our study that CAPE might be a promising compound against CDDP-induced renal failure and oxidative renal damage. In another study using the rat model of CDDP-induced nephrotoxicity [35], our colleagues investigated liver toxicity of rats after CDDP administration. They found an elevation of the NO level and the XO activity and a decrease in the CAT and SOD activities by CDDP, whereas CAPE led to a decrease in NO and XO, as well as an increase in CAT and SOD activities in liver tissue. This shows that CAPE significantly prevents the CDDP toxicity in the liver tissue.

Doxorubicin (DXR)-induced toxicity is known to be mediated through several mechanisms including ROS formation and iron-based oxidative damage to biological macromolecules such as proteins, DNA, RNA, and membrane lipid peroxidation [36, 37]. The effect of CAPE on tissue lipid peroxidation, antioxidant status, and NO levels in DXR-induced nephrotoxicity was investigated by Yagmurca et al. [8]. According to their findings, they demonstrated that a 20 mg/kg single injection of DXR to the Sprague-Dawley rats caused renal injury including glomerular and tubular lesions 10 days after the DXR injection. However, pretreatment with CAPE protected renal tissue against DXR-induced nephrotoxicity according to biochemical and histopathological criteria.

Methotrexate (MTX) is a well-known cytotoxic antimetabolite commonly used in high doses in certain types of cancer of the breast, skin, lung, head, and neck. It is also used to treat severe psoriasis and rheumatoid arthritis (RA). RA is caused by excessive activity of the immune system; MTX may work by suppressing this excessive activity and so reducing inflammation. In psoriasis, MTX prevents the excessive division and multiplication of skin cells that causes skin scaling and raised plaques. In the case of cancer, the cytotoxic properties of MTX are not selective for cancer cells but also affect normal tissues that have a high rate of proliferation, including hematopoietic cells of the bone marrow and the actively dividing cells of gut mucosa [38]. Moreover, MTX has been shown to have potential side effects on many organs, particularly on the liver and kidney [39, 40]. It is known that MTX produces ROS and thus causes lipid peroxidation by affecting the lipid components of the cell membrane, leading to mitochondrial function impairment [41]. Long-term uses of MTX may lead to acute renal failure [42]. The effects of CAPE treatment against MTX-induced injury on kidney were investigated by means of biochemistry and histopathology. Authors analyzed MDA, GSH, MPO, Na/K-ATPase, TNF-*α*, and IL-*β* and applied microscopic evaluation [43]. Serum TNF-*α* and IL-1*β* were found to be increased in MTX-treated rats but the levels of these molecules decreased with CAPE application. GSH and Na/K-ATPase levels were decreased but MPO and MDA levels were increased upon MTX administration, whereas CAPE caused a sharp normalization of these three parameters. Glomerular congestion and degeneration, as well as dilatation in Bowman's space and tubular degeneration, were noticed in MTX-applied group of rats; however, CAPE application changed all the pathologies into normal kidney parenchyma and regular morphology. As a summary, the findings of the study illustrate that CAPE is capable of reducing MTX-induced renal injury possibly through its anti-inflammatory and antioxidant effects, which were evaluated both biochemically and histologically. Kidney seems to demonstrate a better tolerance to MTX treatment when CAPE is used as protective agent. Again, the subject was investigated by our colleagues in Isparta by using Wistar albino rats [44]. A couple of oxidant and antioxidant parameters in terms of MDA, as well as Cu, Zn- and Mn-SOD, CAT, and GSH-Px, were analyzed in kidney homogenates after MTX and MTX plus CAPE were applied. All the antioxidant enzyme activities were found to be decreased upon MTX application whereas CAPE prevented this decrease in antioxidant enzyme activities, a finding that clarifies the questions about the mechanisms. Moreover, CAPE caused a decrease in lipid peroxidation end products, MDA after a sharp increase by MTX administration. Our colleagues explained this protective effect of CAPE by attributing its scavenging activity for ROS in kidney tissue. The same research team did another study by changing the parameters investigated [45]. In this case, they analyzed NO, ADA, and XO in renal tissues in rats subjected to MTX 20 mg/kg i.p. as a single dose. While XO and ADA activities increased in MTX group, NO level was found to be decreased. CAPE administration reversed both the increase of XO and ADA and the decrease in NO level in kidney tissue.

### 2.4. Gentamycin, Vancomycin, and Amikacin Nephrotoxicity

Gentamycin, an antibiotic of the aminoglycoside class, is used to treat many types of bacterial infections. The ototoxic and nephrotoxic adverse effects limit the usage of this antibiotic extensively. The toxic effects of this antibiotic are originated from its inhibitory effect on protein synthesis in renal cells. This mechanism usually causes cell necrosis in the proximal tubule resulting in acute tubular necrosis, which ultimately can lead to acute renal failure [46]. Aygün et al. hypothesized that the suppression of immune cells with CAPE might cause a decrease in ROS production and thus decided to investigate whether the status of gentamycin-induced rat kidney could be favorably affected by CAPE treatment [47]. Serum BUN and creatinine levels were found to be elevated in the gentamycin group of rats although their levels were significantly lower in the gentamycin plus CAPE group. On the other hand, renal MDA and NO levels were higher and CAT, SOD, and GSH-Px activities were lower in the gentamicin group. CAPE application restored the parameters to their normal values. In parallel with these findings, the authors observed tubular necrosis, tubular dilatation, tubular epithelial desquamation, tubular vacuolization, tubular slender, intestinal edema, necrotic areas in the cortex, mononuclear cells in medulla, and hyperchromatic nuclei in the kidneys of the gentamycin group, whereas there was no change in the kidney of rats treated with CAPE. Vardi et al. observed the effects of gentamycin on lipid peroxidation and renal histology and investigated the possible protective effect of CAPE against gentamycin-induced renal damage in rats [48]. MDA level was found to be significantly increased in the renal tissue of gentamycin-treated rats and coadministration of CAPE with gentamycin normalized the MDA level. In the light microscopic examination, gentamycin-treated rats showed marked tubular necrosis and desquamation of the cortical tubular epithelial and also gentamycin induced a marked apoptotic reaction in proximal tubules. Moreover, basal membrane interruption and loss of the brush border were also observed with PAS staining in the affected proximal tubules. On the other hand, the tubules from rats of the gentamycin plus CAPE group were almost normal in histological appearance except for slight desquamations and atrophies of the tubular epithelial cells. Tubular injury was shown to be markedly reduced in the gentamycin plus CAPE group. Both biochemical and histological findings evidenced that coadministration of CAPE together with gentamycin has the capability to prevent nephrotoxicity. In another study, female Wistar rats were used to evaluate gentamycin-induced renal damage and the potential protection of CAPE [46]. Serum creatinine, BUN, tissue SOD, CAT, GSH, NO, MDA, and histological analysis were performed in rat groups. Again, as in the previous studies, the increase in serum creatinine and BUN levels induced by gentamycin was significantly blocked by CAPE. Gentamycin administration to rats caused increases in renal MDA and NO but decreases in SOD and CAT activities as well as GSH content. CAPE administration together with gentamycin caused significant decreases in MDA and NO levels and significant increases in SOD, CAT, and GSH. Histological data supported the biochemical findings, namely, gentamycin caused marked and extensive tubular necrosis but CAPE administration blocked kidney tissue damage as measured semiquantitatively.

Vancomycin, as a structurally glycopeptide antibiotic, has been used for Gram-positive bacteria, especially in methicillin-resistant* Staphylococcus aureus* (MRA) infections. It is a naturally occurring antibiotic made by soil bacterium. It inhibits proper cell wall synthesis in bacteria. Damage to the kidney and damage to the hearing system are frequent side effects of vancomycin [49]. The use of more pure forms of vancomycin decreases nephrotoxicity but coadministration of aminoglycosides may accentuate this adverse effect [50]. Nephrotoxicity associated with continuous vancomycin infusion is not rare, should not be underestimated, and should be dealt with seriously [51, 52]. In a review article for the strategies supporting the prevention of harmful effects of vancomycin to the kidney, the use of antioxidant agents including CAPE for vancomycin nephrotoxicity was discussed [53]. Ocak et al. designed a study to investigate the beneficial effects of CAPE, N-acetylcysteine, vitamin C, and vitamin E on vancomycin-induced nephrotoxicity in rats [54]. After vancomycin injection (200 mg/kg, i.p., single dose), which is known to induce much enough nephrotoxicity [55], CAPE (10 *μ*mol/kg, i.p.) was applied for the following 7 days with other protective molecules in each rat group separately. Serum BUN concentration was decreased by CAPE administration, although there was a significant increase caused by vancomycin. Increased renal MDA and NO concentrations in vancomycin-applied rats were significantly suppressed by CAPE. Histopathological examination revealed tubular necrosis, degeneration, vacuolization, interstitial edema, tubular atrophy, and inflammatory cell infiltration upon vancomycin administration. All these damages were reversed by CAPE.

Amikacin, a member of aminoglycosides antibiotics, is used for Gram-negative infections. Although it has high antibacterial efficacy, low rate of resistance, synergistic effects with *β*-lactam antibiotics, and low cost, the nephrotoxic side effects limit its safe use. The damaging effects on kidney are attributed to generation of ROS [56, 57]. Based on this information, a study was designed in rats to investigate the effects of CAPE as a single dose on amikacin-induced histopathological changes and biochemical parameters including MDA, BUN, and creatinine [58]. In the corresponding groups of rats, amikacin was administered at a single dose i.p. 1.2 g/kg and CAPE at 10 *μ*mol/kg starting with 1 hour before amikacin injection during two days up to sacrification of rats. Amikacin had a minimal effect on BUN and creatinine but tissue MDA levels were doubled. CAPE led to a decrease in MDA levels. Morphological damage ranged from none in the control group to mild in the CAPE group and to severe in the amikacin group, suggesting that CAPE is an available agent to protect renal tissues from amikacin-induced oxidative damage.

### 2.5. Cadmium Nephrotoxicity

As a heavy metal, cadmium (Cd) and its compounds are toxic. The most dangerous form of occupational exposure to Cd is inhalation of fine dust and fumes, or ingestion. After entering the body, the highest concentration of Cd has been found to be absorbed in the human kidney. Throughout childhood and adolescence, the inhaled Cd was estimated to be about 30 mg [59]. The energy producing organelles, the mitochondria, are known to be key intracellular targets of Cd. Mitochondrial dysfunction has an impact on the health of cells via mechanisms involving increased ROS production [60–62]. Cd can cause chronic kidney disease and end-stage renal disease after heavy exposure [61]. If medical strategies preserve mitochondria from ROS attack, then the strategy might be successful to preserve normal kidney functioning. Kobroob et al. decided to address this issue using isolated rat kidney mitochondria exposed to Cd as an experimental* in vitro* model [63]. After systemic perfusion and sacrifice, rat kidneys were excised and the mitochondrial fraction was prepared. Isolated kidney mitochondria were incubated with 0, 10, 20, 30, and 40 *μ*M of cadmium chloride (CdCl_2_) for 5 min. The changes in mitochondrial functions were evaluated by detection of swelling, ROS production, and membrane potentials of mitochondria. CAPE groups were applied 0.1, 1, and 10 of *μ*M CAPE 10 min before Cd administration and all the functions mentioned above were measured again. The results of this study provided evidence for the mitochondrial protective effects of CAPE and suggested that this beneficial effect is mediated by its ROS scavenging potential that eventually prevents oxidative stress in mitochondria. Interestingly, Cd-induced kidney mitochondrial dysfunction was accompanied by a substantial rise in mitochondrial ROS, NO, and MDA levels and a decrease in SOD activity. Pretreatment of mitochondrial tissue homogenate with CAPE both ameliorated mitochondrial dysfunction and restored all the changes in oxidative parameters mentioned above. Although all doses of CAPE used seemed to be advantageous, the greatest therapeutic benefit was apparent at 10 *μ*M concentration. CAPE was not totally successful for every aspect of the analyzed parameters for mitochondria, because it can only partially reduce the swelling of mitochondria. Authors claimed that ROS-induced mitochondrial permeability transition pore opening might not be the only mechanism underlying mitochondrial swelling triggered by calcium. In the light of previous data, this study provided more evidence to reinforce the promising antioxidant role for CAPE to combat renal toxicity of Cd.

To explain the mechanisms of Cd-induced renal toxicity and to investigate the beneficial effect of CAPE on Cd-induced renal damage, Gong et al. planned an experimental study using male adult Kunming mice [64]. Because the LD50 for a single i.p. dose of Cd in mice is 6.75 mg/kg body weight, the animals were given 1 mg/kg CdCl_2_ together with protective agent CAPE as 0.1, 1, and 10 *μ*mol/kg body weight in corresponding rat groups. Researchers analyzed protein content, metallothionein amount, trace element concentration, and oxidative stress and antioxidant defense system parameters in kidney tissue preparations. Cd administration elevated lipid peroxidation and protein carbonyl as well as CAT activity. It also decreased renal content of SOD and GSH. Upon CAPE administration, all the changes in oxidative and antioxidant parameters reversed to more or less control levels. The authors proposed an underlying mechanism associated with CAPE's antioxidant capacity, anti-inflammatory effects, and the capacity to alter NF*κ*B expression via activation of Nrf2 pathway. Metallothionein was not found to be involved in the protective action of CAPE on Cd-induced renal damage.

### 2.6. Miscellaneous Nephrotoxicities and Injuries by Several Other Factors

*Lithium* (Li) has been used for some diseases such as bipolar disorder and has been known to have toxic effects on several biological systems, including the kidney [65]. In long-term usage, it may induce progressive nephrotoxicity [66]. The mechanism of Li toxicity is still poorly understood. There are some hypotheses based on ROS-triggered tissue injury. The renal tubular effects and oxidative stress in kidney after long-term Li use and also the protective effect of CAPE were investigated in a rat model [67]. Rats were administered Li 25 mg/kg i.p. as Li_2_CO_3_ twice daily for 4 weeks. CAPE was coadministered i.p. once a day at a dose of 10 *μ*mol/kg for 4 weeks. Both MDA and urinary N-acetyl-beta-D-glucosaminidase/creatinine ratio were increased in Li administration and upon CAPE treatment they were decreased to control levels. SOD, CAT, and GSH-Px activities in the kidney tissue were found to be decreased in Li-applied rats, whereas upon CAPE treatment they were kept in a level similar to that of the control group of rats. CAPE also reduced Li-induced oxidative stress-mediated renal tubular damage in renal tissues.

*Toluene* is widely used in industries where adhesives, erasers, plastics, leather, dyes, dye thinners, and printing pastes are manufactured. It has an addictive potential as well. That is why substance abuse is a matter of concern. It has lipophilic properties and easily diffuses through tissues and generates toxic effects in certain organs such as kidney. Protective effect of CAPE was studied against the toxic effect of toluene [68]. Intraperitoneally injected toluene and toluene plus CAPE were investigated in terms of kidney toxicity. MDA, SOD, CAT, and GSH-Px activities, as well as serum creatinine and BUN analysis, and also histological examination, were carried out. In brief, as an antioxidant, CAPE was found to protect kidney from renal damage.

*Carbon tetrachloride* (CCl_4_) is also a toxic liquid that has been used for experimental hepatic cirrhosis as well as an organic solvent. Besides its toxic effect on liver, it is also known to be toxic for the kidney. Several natural antioxidants including vitamins C and E, ginkgo biloba, black tea extract, and others were reported to prevent or reduce CCl_4_-induced nephrotoxicity [69, 70]. Lately, CAPE was used for the treatment of CCl_4_-induced renal damage [71]. Rats were injected 0.5 mL/kg body weight CCl_4_ every other day for one month and 10 *μ*mol/kg body weight CAPE to assigned groups. As in other previous papers, the authors analyzed serum urea and creatinine, tissue MDA as well as histopathological analysis. They found serum urea and creatinine to be increased upon CCl_4_ administration and that CAPE treatment normalized these two parameters related to renal function. Tissue MDA levels were decreased by CAPE administration after a sharp increase induced by CCl_4_. In CCl_4_-treated kidneys, the seriously affected glomeruli in addition to the renal corpuscles with normal appearance exhibit different forms of degenerations together. Some glomeruli were shown to have mild dilatation of Bowman's space with glomerular atrophy. In the CAPE group, interstitial inflammatory cell infiltrations were absent, and histological appearances of the glomeruli and tubules were normal. Furthermore, the increase in the connective tissue seen in CCl_4_ infusion was not observed after CAPE treatment.

There has been substantial evidence for the notion that, when ingested in toxic doses,* acetylsalicylic acid (ASA)* can induce two different forms of acute renal injury—hemodynamically mediated and acute interstitial nephritis—that are directly related to a reduction in prostaglandin synthesis [72, 73]. By supposing that compounds that act as antioxidants may contribute to the improvement of ASA-mediated renal toxicity, researchers aimed to investigate the protective effects of CAPE against ASA-induced renal oxidative stress used as readouts biochemical parameters and the histopathology of kidney [68]. Rats were given ASA at dose of 50 or 100 mg/kg/day for 5 days via orogastric gavage or ASA together with CAPE at dose of 20 *μ*g/kg/day for 5 days. The tissue specimens and serum samples were analyzed for total oxidant capacity (TAC), total oxidant status (TOS), and paraoxonase-1 (PON-1) activities, and of course for histopathology using light microscopy. The TOS levels in the serum and kidneys were increased in rats given ASA but the levels of TAC and PON-1 in the kidney tissue decreased significantly. Upon CAPE administration, TOS level was decreased in kidney tissue. The PON-1, TAC, and TOS values were reverted to normal values being supported by histopathological observation.

*Paraquat*, a potent herbicide, has been known to show its toxic effects due to free oxygen radicals. Unfortunately, no antidote or effective treatment exists for paraquat intoxication. Therefore, several studies have been conducted to find an available agent to prevent paraquat toxicity. Rifaioglu et al. investigated paraquat-induced biochemical and histological changes in kidney, one of main targets of paraquat, and the possible protective effects of CAPE in rats [74]. By measuring two important ROS-related parameters, total antioxidant capacity and total oxidant status, as well as histologic scores of affected kidneys, they found that CAPE can be used to prevent the acute effects of paraquat nephrotoxicity.

An* in vivo* model of myoglobinuric acute renal failure is the injection of* hypertonic glycerol* into muscles, which in turn causes myolysis, hemolysis, and intravascular volume depletion and exposes the kidney to a large burden of heme proteins, myoglobin, and hemoglobin leading to tubular nephropathy by the generation of ROS [75, 76]. Assuming that, as an antioxidant, CAPE would be a potential protective candidate for glycerol-induced renal failure because of the oxidative nature of physiopathology, researchers conducted a study using Wistar albino rats and analyzed renal tissues after a high dose of glycerol injection to the muscles (10 mL/kg, single shot) [77]. CAPE was applied i.p. 10 *μ*mol/kg body weight. Renal functions and oxidative status of kidney from the point of both biochemical and histological views were assessed. Although SOD activity in the CAPE group was found to be increased, they failed to find any positive effect of CAPE on the parameters studied. However, increased plasma urea and MDA, decreased plasma NO, and a quite high death rate of rats were found in the CAPE group, and the worsening effect of CAPE was attributed to the depletion of NO by CAPE. The other mechanism suggested was the resultant possible renal vasoconstriction, which was not measured by the authors, leading to severe renal ischemia.

*Tobacco* contains several carcinogenic substances and these substances are mostly removed by the kidney after being metabolized in the liver. They can induce or manage progression of renal diseases including glomerulosclerosis, end-stage renal failure dependent on inflammatory and noninflammatory renal diseases, and renal-cell carcinoma [65, 78]. The ameliorative effect of CAPE administration on nephrotoxicity caused by cigarette smoking was investigated using Wistar albino rats [79]. Two cigarettes lit for 30 min in each period were placed in cages four times a day during a 60-day experiment; in the CAPE group of rats, CAPE was injected in a dose of 10 *μ*mol/kg, i.p. daily throughout the study. The light microscopic evaluation of tissue samples from kidney of rats exposed to cigarette smoke revealed mesangial cell proliferation in the renal corpuscle, dilatation and congestion in the peritubular capillaries, degeneration in the proximal tubules and atrophic renal corpuscle. These histopathological changes were partially cured in rats exposed to cigarette and CAPE. Serum uric acid and BUN levels were elevated in the smoking group, while no changes noted in the CAPE group. Moreover, increased levels of renal SOD, GSH-Px, NO, and MDA were obtained by CAPE administration, showing the protective effect of CAPE on the harmful effect of smoking on kidneys.

*Age* related physiological changes in the kidney are both structural and functional. The oxidative stress that tends to be increased by age [80] has become the prominent theory to explain the effect of aging at the molecular level. Morphological changes in kidney involve the renal blood vessels, glomeruli, tubules and interstitium [81] showing thickening of the intrarenal vascular intima, infiltration of inflammatory cells, fibrosis in the stroma and the structural changes in glomeruli. Researchers investigated the kidneys from old rats by using light and electron microscopes and the effect of CAPE administration [82]. The young rat group (4-month old) and the older rat group (18-month old) were compared, receiving (i.p. 15 mg/kg/day for 95 days)/not receiving CAPE. Tissue MDA level was increased whereas GSH level and SOD, CAT, and GSH-Px activities were decreased by age. CAPE significantly reduced tissue MDA levels but increased tissue SOD, CAT and GSH-Px activities and GSH levels. The mean semi-quantitative damage score of old rats was found to be statistically higher than those of young ones. CAPE administration significantly reduced histopathological changes seen under the light microscope (reduced tubular and epithelial degeneration, sclerosis, cell infiltration, thyroidization, and tubular dilatation). In the electron microscope evaluation, tubular and glomerular components were normal in young animals whereas the most prominent aging-induced alterations were edema, vacuole formation, lysosome and lipofucsin accumulation within the tubular cells, and thickening of the tubular basement membranes. Besides, mitochondrial degenerative changes such as edema, vacuole formation, cristae loss, or thickening of cristae membranes were observed. Tubular cells were irregular in shape; microvillus loss and disorganization were obvious. Glomerular basement membranes were also thickened. Tubular and glomerular structures were well preserved in CAPE-administered rats. However, rare vacuole formation in CAPE group was observed.

*Thermal injury* is another strong event leading to hypovolemia and I/R in distant organs such as kidney. The changes in kidney start as a chain reaction such as sequestration of polymorphonuclear leukocytes, activation of neutrophils and XO system, metabolism of arachidonic acid, release of free metal ions that have redox potential which in turn leads to OH^∙^ production from H_2_O_2_ via Fenton reaction, release of inflammatory cytokines, platelet aggregation and other metabolic changes [83]. Gurel et al. examined the effects of CAPE treatment on kidney tissue after thermal injury in an animal model [84]. For this purpose, they determined MDA, SOD, CAT, XO, MPO activities and levels after the 1st, 3rd, 7th day of to post-burn period. Severe skin thermal injury caused a significant decrease in SOD and CAT activities as well as a significant increase in MDA level, XO and MPO activities in kidney during the post-burn period. Treatment of rats with CAPE significantly elevated SOD and CAT activities while it caused a decrease in MDA levels as well as MPO and XO activities.

The close proximity of the 900 MHz* electromagnetic radiation* (EMR) emitting mobile phones of the abdominal organs when carried on the belt has raised concerns about the biological interactions between EMR and the kidney [85]. The role of ROS has been implicated in mobile phone-based oxidative injury in several organs [86–88]. The rats were exposed to 900 MHz EMR emitted mobile phone 30 min/day for 10 days using experimental phone exposure device and a group of rats was pretreated with CAPE at a dose of 10 *μ*mol/kg/day starting from 10 days before phone exposure [89]. The lysosomal enzyme N-acetyl-*β*-D-glucosaminidase (NAG) in urine as well as lipid peroxidation end products and antioxidant enzymes in kidney tissue were evaluated in the tissue after rats were decapitated. Urinary NAG and renal MDA were increased in EMR-exposed rats while CAPE caused a significant reduction in these parameters. On the other hand, renal SOD and GSH-Px activities were decreased in EMR exposed animals but CAPE did not affect these enzyme activities and they were retained at this diminished level. The authors concluded that CAPE was a promising agent against tubular injury by reducing oxidative stress and protecting the kidney from oxidative damage induced by 900 MHz mobile phone; nevertheless, melatonin was also tested in that experiment and found to be more potent than CAPE. The same research team conducted another study using the same experimental design but the exposure time was determined as 3 months instead of 10 days [90]. According to the findings, in the EMR group, while tissue MDA, NO and urinary NAC levels increased, SOD, CAT and GSH-Px activities reduced. CAPE reversed these effects and normalized the parameters' levels.

*Extracorporeal shock wave lithotripsy* (ESWL) is a noninvasive routine treatment for urolithiasis but it has some side effect possibly because ROS formation after thermal effects of 18–24 thousand volts affecting macromolecules [91, 92]. The potential protective effect of CAPE on the shock wave-induced oxidative stress in rabbit kidney was investigated [93] in which the animals were exposed to 3000 shock waves at 18 kV under intramuscular ketamine anesthesia. Both ESWL and ESWL + CAPE groups were exposed to shock waves for 10 days and CAPE was injected daily at 10 *μ*mol/kg dose, i.p. Shock wave exposure significantly increased the levels of MDA, urine NAG activity, uric acid and white cell count in renal tissue, whereas CAPE prevented all the changes in these parameters attributing the free radical scavenger activity of this compound.

*Radio-contrast media* may cause renal insufficiency in the high-risk population including patients with renal impairment, diabetes mellitus, congestive heart failure, and elderly patients [94]. Studies about contrast-induced nephropathy have been focused on the mechanism and prevention. Authors hypothesized that CAPE may have comparable protective effect on kidney against contrast nephropathy induced by hypoxia, ROS and direct tubular toxicity [95]. The study group received 7 mL/kg diatrizoate and the CAPE group received 50 *μ*mol/kg CAPE, i.p., plus diatrizoate for three days. Creatinine, oxidant/antioxidant status as renal histopathology were examined and found that the levels of MDA and antioxidant enzymes were high and low, respectively, in renal tissue exposed to contrast material showing increased lipid peroxidation. CAPE has shown a renal protective effect against contrast material-induced functional and structural nephropathy. Renal histopathological examination of the control group showed no pathological findings whereas glomerular injury, tubular vacuolization-necrosis, interstitial edema and interstitial infiltration scores were significantly higher in the contrast material group. Although minimal histopathological changes detected in the CAPE group, renal injury scores were not different from those of the control group. As a result, researchers suggested that CAPE might be accepted as a protective agent for renal structure and functions in contrast material injury.

## 3. Conclusion

We first critically evaluated and summarized the protective effect of CAPE in nephrotoxicity induced by several compounds such as cisplatin, doxorubicin, cyclosporine, gentamycin, methotrexate, amikacin, lithium, toluene, carbon tetrachloride, acetylsalicylic acid, and glycerol using pathological evaluation and measurement of some oxidant and antioxidant biochemical parameters mostly in rats (Figure 3). In addition, we summarized the effect of CAPE on thermal trauma, aging, mobile phone-induced renal impairment, contrast nephropathy, extracorporeal shock wave lithotripsy, and renal I/R injury. The protective effect of CAPE on the above-mentioned situations might be due to the inhibition of leukocyte accumulation in the kidney, the scavenging of ROS by CAPE itself, and/or the promotion of the antioxidant enzyme activities (Figure 3). Our data suggest that CAPE might be a promising new therapeutic agent for all kinds of nephrotoxicity and oxidative renal damage.

## Figures and Tables

**Table 1:** *In vivo* studies showing several types of action, animals recruited, doses applied, and reported outcomes of CAPE in some disease models of several organs except kidney.

The type of study	Organ	Animals used	The dose applied	Type of action	Reported outcomes	Reference
Melanoma tumor model	Skin	C57BL/6 mice	10, 20, 30 mg/kg/day	Increases ROS	Antimelanoma efficacy	[96]

Colorectal adenocarcinoma model	Colon	BALB/c mice	10 mg/kg, i.v.	Depletes GSH and inhibits NF*κ*B activity	Sensitizes CT26 colorectal adenocarcinoma to ionizing radiation	[97]

Short-term myocardial I/R	Heart	Albino Wistar rats	10 *μ*mol/kg, i.p.	A combination of decreased XO activity and direct antioxidant effect	Cardioprotective effect	[98]

Long-term myocardial I/R	Heart	Albino Wistar rats	10 *μ*mol/kg, i.p.	Reduces apoptosis and serum CK and AST activities, attenuates NO production, elevates myocardial SOD	Pretreatment with CAPE provides cardioprotective effects	[99]

Cisplatin-induced oxidative damage	Liver	Albino Wistar rats	10 *μ*mol/kg/day, i.p.	Strengthens the antioxidant defence system by reducing ROS and increasing antioxidant enzyme activities	Prevents cisplatin-induced oxidative changes in liver	[100]

Pentylenetetrazol-induced seizure	Brain	Swiss albino female mice	10 *μ*mol/kg, i.p.	Decreases MDA and NO levels by its antioxidative action	Protects the brain from PTZ-induced oxidative damage	[101]

Ototoxicity induced by cisplatin	Blood	Albino Wistar rats	10 *μ*mol/kg/day, i.p.	Decrease in XO activity	Ameliorates hearing deterioration	[102]

Experimental autoimmune encephalomyelitis	Spinal cord and brain	Inbred female Wistar rats	25 *μ*mol/kg/day, i.p.	Anti-inflammatory activity	Protects the CNS from oxidative damage	[103]

Bleomycin-induced lung fibrosis	Lung	Sprague Dawley rats	10 *μ*mol/kg/day, i.p.	Antioxidant and free radical scavenger activities	Protects lungs via decreased hydroxyproline, NO, MDA and MPO levels, increased SOD and CAT activities	[104]

Testicular torsion/detorsion	Testis	Male albino Wistar rats	10 *μ*mol/kg, i.p.	Antioxidant and antineutrophil effects	Attenuates testicular injury histologically and biochemically (decreased MPO and TBARS levels, increased GSH-Px activity)	[105]

Caustic esophageal stricture	Esophagus	Albino Wistar rats	10 *μ*mol/kg/day, s.c.	Supports wound healing by anti-inflammatory, immunomodulatory, and antioxidant properties	Prevents caustic esophageal strictures	[106]

Testicular torsion/detorsion	Testis	Male albino Wistar rats	10 *μ*mol/kg, i.p.	Increases testicular NO levels in ipsilateral, but not in contralateral testes	Protects testes from torsion/detorsion injuries	[107]

Spinal cord I/R injury	Spinal cord	New Zealand white rabbits	10 *μ*mol/kg, i.p.	Scavenges free radicals/provides better microcirculatory environment during reperfusion via preventing endothelial cell lysis by proteases from activated PMNL	Reduces I/R damage in transient spinal cord ischemia and provide better neurologic outcome	[23]

Intestinal reperfusion injury	Intestine	Albino Wistar rats	10 *μ*mol/kg i.p.	Eliminates oxygen radicals and inhibits PMNL infiltration	Prevents reperfusion injuries in intestinal tissue	[24]

AST: aspartate transaminase CAT: catalase, CAPE: caffeic acid phenethyl ester, CK: creatine kinase, CNS: central nervous system, GSH: glutathione, GSH-Px: glutathione peroxidase, i.p.: intraperitoneal, I/R: ischemia/reperfusion, i.v.: intravenous, MDA: malondialdehyde, MPO: myeloperoxidase, NF*κ*B: nuclear factor kappa B, NO: nitric oxide, PMNL: polymorphonuclear leukocytes, p.o.: per oral, PTZ: pentylenetetrazol, ROS: reactive oxygen species, s.c.: subcutanaeous, SOD: superoxide dismutase, TBARS: thiobarbituric acid reactant substances XO: xanthine oxidase.

**Table 2:** *In vivo* studies showing several types of action, animals recruited, doses applied, and reported outcomes of CAPE in some disease models of kidney.

The type of study	Target organ	Animals used	The dose applied	Type of action	Reported outcomes	Reference
Renal I/R model	Kidney	Albino Wistar rats	10 *μ*mol/kg, i.p.	Inhibits neutrophil sequestration	Attenuation in renal damage after I/R	[6]

Renal I/R model	Kidney	Albino Wistar rats	10 *μ*mol/kg, i.p.	Alters the indices of oxidative stress	Partially alleviates renal damage after I/R	[5]

Renal I/R model	Kidney	Albino Wistar rats	10 *μ*mol/kg, i.p.	Suppressed I/R-induced renal lipid peroxidation	Therapeutic advantage in acute injury setting	[19]

Cisplatin-induced nephrotoxicity	Kidney	Female albino Wistar rats	10 *μ*mol/kg/day, i.p.	Free oxygen radical scavenging activity	Marked reduction in the extent of tubular damage	[7]

Paraquat-induced acute nephrotoxicity	Kidney	Female albino Wistar rats	10 *μ*mol/kg, i.p.	Attenuates the oxidative stress caused by paraquat	Protects acute nephrotoxicity induced by paraquat	[74]

Acute urogenital injury following pneumoperitoneum	Kidney, testis, prostate	Albino Wistar rats	10 *μ*mol/kg, i.p.	Affects TAC and TOS levels	Prevents adverse effects of intra-abdominal pressure on kidney and testis	[108]

Toluene-induced nephrotoxicity	Kidney	Male albino Wistar rats	10 *μ*mol/kg/day, i.p.	By showing antioxidant, antitoxic, and nephroprotective effect	Prevents renal damage	[109]

Cadmium-induced kidney mitochondrial injury	Kidney	Male Wistar rats	10 *μ*M final conc. to the isolated mitochondria	Antioxidant potential	Has therapeutic benefits in the setting of nephrotoxicity caused by cadmium	[63]

Acetylsalicylic acid toxicity	Kidney	Albino Wistar rats	20 *μ*g/kg/day, p.o.	Reduces the concentration of oxidant products and supports the antioxidant system	Protects kidneys from ASA-induced nephrotoxicity	[68]

Aging-related oxidative damage	Kidney	Sprague Dawley rats	15 mg/bw/day, i.p.	Antioxidant and high cellular protective effects	Beneficial in delaying age-related cellular changes	[82]

Renal I/R	Kidney	Male Wistar rats	10 *μ*mol/kg, i.p.	Antioxidant effect	Promotes greater functional and anatomic renal injury	[28]

Cd-induced renal damage	Kidney	Adult Cumming mice	0.1 and 1 *μ*mol/kg/day, i.p.	Reduces the levels of oxidative stress and altering the antioxidant defense system	Protects the oxidative renal damage induced by Cd in a dose-dependent manner	[64]

Methotrexate-induced hepatorenal oxidative injury	Kidney	Both sexes albino Wistar rats	10 *μ*mol/kg/day, i.p. for 5 days	Anti-inflammatory and antioxidant effects	Capable of reducing methotrexate-induced hepatorenal oxidative injury	[43]

Gentamycin-induced oxidative nephrotoxicity	Kidney	Female albino Wistar rats	10 *μ*mol/kg/day, i.p. for 12 days	Modulator effect on oxidative stress and antioxidant redox system	Nephrotoxicity may be significantly reduced	[47]

Renal dysfunction by cyclosporine A	Kidney	Male Wistar rats	10 and 30 *μ*mol/kg, i.p. for 10 days	Inhibits renal lipid peroxidation and enhances and maintaining the antioxidant GSH content	Protects against cyclosporine A nephrotoxicity	[33]

Cyclosporine A-induced nephrotoxicity	Kidney	Female albino Wistar rats	10 *μ*mol/kg/day, i.p. for 11 days	Inhibits lipid peroxidation via inhibition of oxidative process	Protects kidney from cyclosporine A-induced damage	[34]

Vancomycin-induced nephrotoxicity	Kidney	Male albino Wistar rats	10 *μ*mol/kg/day, i.p. for 7 days	Decreases lipid peroxidation and increases antioxidant enzyme activity	Reduction of the nephrotoxic effects of vancomycin	[54]

Methotrexate-induced renal oxidative stress	Kidney	Albino Wistar rats	10 *μ*mol/kg/day, i.p. for 7 days	Shows a potent scavenging effect of free radicals	Reduces renal impairment	[44]

Lithium-induced renal toxicity	Kidney	Male albino Wistar rats	10 *μ*mol/kg/day, i.p. for 4 weeks	Significant increase in the activities of antioxidant enzymes and decrease in lipid peroxidation	Reduces Li-induced oxidative stress mediated renal tubular damage	[44]

Long-term mobile phone exposure/renal impairment	Kidney	Male Sprague-Dawley rats	10 *μ*mol/kg/day, i.p. for 3 months	Free radical scavenging and antioxidant properties	Protects renal tissue from oxidative damage and prevents organ impairment	[90]

Amikacin-induced nephrotoxicity	Kidney	Female Wistar rats	10 *μ*mol/kg/day, i.p. for 2 days	Decreases MDA levels showing lipid peroxidation-preventive effects	Protects kidney tissue against oxidative damage	[58]

Shock wave-induced renal tubular oxidative stress	Kidney	White rabbits	10 *μ*mol/kg/day, i.p. for 10 days	Reduces significantly MDA levels, urine NAG activity, uric acid and white cell count in renal tissue	Avoiding the side effects of ESWL applications	[93]

Carbon tetrachloride-induced renal toxicity	Kidney	Male albino Wistar rats	10 *μ*mol/kg, i.p. for every other day for one month	Reduces MDA levels by antioxidant properties	Protective effect on CCl_4_-induced kidney damage	[71]

Oxidative organ damage due to thermal trauma	Kidney	Male albino Wistar rats	10 *μ*mol/kg/day, i.p. for max. 7 days	Scavenges free oxygen radicals, decreases MPO activity in neutrophils, increases antioxidant enzyme	A potential beneficial agent in humans who suffer from thermal injury	[84]

Doxorubicin-induced nephrotoxicity	Kidney	Male Sprague-Dawley rats	10 *μ*mol/kg/body weight/day, i.p. for 12 days	Antioxidant and anti-inflammatory effects	Protects renal tissues against DXR-induced toxicity	[8]

ASA: acetylsalicylic acid, CAPE: caffeic acid phenethyl ester, CCl_4_: carbon tetrachloride, Cd: cadmium, DXR: doxorubicin, ESWL: extracorporeal shock wave lithotripsy, GSH: glutathione, i.p.: intraperitoneal, I/R: ischemia/reperfusion, Li: lithium, MDA: malondialdehyde, MPO: myeloperoxidase, NAG: N-acetyl-*β*-D-glucosaminidase, p.o.: per oral, TAC: total antioxidant capacity, TOS: total oxidant status.

## Abbreviations

ADA: Adenosine deaminase
BUN: Blood urea nitrogen
CAPE: Caffeic acid phenethyl ester
CAT: Catalase
CCl_4_: Carbon tetrachloride
Cd: Cadmium
CdCl_2_: Cadmium chloride
CDDP: cis-diamminedichloroplatinum
CsA: Cyclosporine
DXR: Doxorubicin
EMR: Electromagnetic radiation
ESWL: Extracorporeal shock wave lithotripsy
GSH-Px: Glutathione peroxidase
GSH: Glutathione
H_2_O_2_: Hydrogen peroxide
I/R: Ischemia/reperfusion
Li: Lithium
MDA: Malondialdehyde
MPO: Myeloperoxidase
MRA: Methicillin-resistant staphylococcus aureus
MTX: Methotrexate
NAG: N-acetyl-*β*-D-glucosaminidase
NO: Nitric oxide
NOS: Nitric oxide synthase
O_2_^∙−^: Superoxide radical
^∙^OH: Hydroxyl radical
PMNLs: Polymorphonuclear leukocytes
PON-1: Paraoxonase-1
RA: Rheumatoid arthritis
ROS: Reactive oxygen species
SOD: Superoxide dismutase
TAC: Total antioxidant capacity
TAS: Total antioxidant status
TGF-*β*: Transforming growth factor-*β*
TOS: Total oxidative status
XO: Xanthine oxidase.

## References

[1] Burdock GA (1998). Review of the biological properties and toxicity of bee propolis (propolis). *Food and Chemical Toxicology*.

[2] Sud’ina GF, Mirzoeva OK, Pushkareva MA, Korshunova GA, Sumbatyan NV, Varfolomeev SD (1993). Caffeic acid phenethyl ester as a lipoxygenase inhibitor with antioxidant properties. *FEBS Letters*.

[3] Watabe M, Hishikawa K, Takayanagi A, Shimizu N, Nakaki T (2004). Caffeic acid phenethyl ester induces apoptosis by inhibition of NF*κ*B and activation of Fas in human breast cancer MCF-7 cells. *Journal of Biological Chemistry*.

[4] McEleny K, Coffey R, Morrissey C, Fitzpatrick JM, Watson RWG (2004). Caffeic acid phenethyl ester-induced PC-3 cell apoptosis is caspase-dependent and mediated through the loss of inhibitors of apoptosis proteins. *BJU International*.

[5] Gurel A, Armutcu F, Sahin S (2004). Protective role of *α*-tocopherol and caffeic acid phenethyl ester on ischemia-reperfusion injury via nitric oxide and myeloperoxidase in rat kidneys. *Clinica Chimica Acta*.

[6] Özyurt H, Kemal Irmak M, Akyol Ö, Söğüt S (2001). Caffeic acid phenethyl ester changes the indices of oxidative stress in serum of rats with renal ischaemia-reperfusion injury. *Cell Biochemistry and Function*.

[7] Özen S, Akyol Ö, Iraz M (2004). Role of caffeic acid phenethyl ester, an active component of propolis, against cisplatin-induced nephrotoxicity in rats. *Journal of Applied Toxicology*.

[8] Yagmurca M, Erdogan H, Iraz M, Songur A, Ucar M, Fadillioglu E (2004). Caffeic acid phenethyl ester as a protective agent against doxorubicin nephrotoxicity in rats. *Clinica Chimica Acta*.

[9] Grunberger D, Banerjee R, Eisinger K (1988). Preferential cytotoxicity on tumor cells by caffeic acid phenethyl ester isolated from propolis. *Experientia*.

[10] Akyol S, Ginis Z, Armutcu F, Ozturk G, Yigitoglu MR, Akyol O (2012). The potential usage of caffeic acid phenethyl ester (CAPE) against chemotherapy-induced and radiotherapy-induced toxicity. *Cell Biochemistry and Function*.

[11] Tolba MF, Esmat A, Al-Abd AM (2013). Caffeic acid phenethyl ester synergistically enhances docetaxel and paclitaxel cytotoxicity in prostate cancer cells. *IUBMB Life*.

[12] Chiao C, Carothers AM, Grunberger D, Solomon G, Preston GA, Barrett JC (1995). Apoptosis and altered redox state induced by caffeic acid phenethyl ester (CAPE) in transformed rat fibroblast cells. *Cancer Research*.

[13] Lee Y-J, Liao P-H, Chen W-K, Yang C-C (2000). Preferential cytotoxicity of caffeic acid phenethyl ester analogues on oral cancer cells. *Cancer Letters*.

[14] Chen Y-J, Shiao M-S, Wang S-Y (2001). The antioxidant caffeic acid phenethyl ester induces apoptosis associated with selective scavenging of hydrogen peroxide in human leukemic HL-60 cells. *Anti-Cancer Drugs*.

[15] Guarini L, Su -z. Z, Zucker S, Lin J, Grunberger D, Fisher PB (1992). Growth inhibition and modulation of antigenic phenotype in human melanoma and glioblastoma multiforme cells by caffeic acid phenethyl ester (CAPE). *Cellular and Molecular Biology*.

[16] Lee Y-J, Kuo H-C, Chu C-Y, Wang C-J, Lin W-C, Tseng T-H (2003). Involvement of tumor suppressor protein p53 and p38 MAPK in caffeic acid phenethyl ester-induced apoptosis of C6 glioma cells. *Biochemical Pharmacology*.

[17] Avci ÇB, Gündüz C, Baran Y (2011). Caffeic acid phenethyl ester triggers apoptosis through induction of loss of mitochondrial membrane potential in CCRF-CEM cells. *Journal of Cancer Research and Clinical Oncology*.

[18] Akyol S, Ozturk G, Ginis Z, Armutcu F, Yigitoglu MR, Akyol O (2013). In vivo and in vitro antineoplastic actions of Caffeic acid phenethyl ester (CAPE): therapeutic perspectives. *Nutrition and Cancer*.

[19] Irmak MK, Koltuksuz U, Kutlu NO (2001). The effect of caffeic acid phenethyl ester on ischemia-reperfusion injury in comparison with *α*-tocopherol in rat kidneys. *Urological Research*.

[20] Kirkebøen KA, Strand OA (1999). The role of nitric oxide in sepsis—an overview. *Acta Anaesthesiologica Scandinavica*.

[21] Walker LM, Walker PD, Imam SZ, Ali SF, Mayeux PR (2000). Evidence for peroxynitrite formation in renal ischemia-reperfusion injury: studies with the inducible nitric oxide synthase inhibitor L-N6-(1-iminoethyl)lysine. *Journal of Pharmacology and Experimental Therapeutics*.

[22] Wangsiripaisan A, Gengaro PE, Nemenoff RA, Ling H, Edelstein CL, Schrier RW (1999). Effect of nitric oxide donors on renal tubular epithelial cell-matrix adhesion. *Kidney International*.

[23] Ilhan A, Koltuksuz U, Ozen S, Uz E, Ciralik H, Akyol O (1999). The effects of caffeic acid phenethyl ester (CAPE) on spinal cord ischemia/reperfusion injury in rabbits. *European Journal of Cardio-Thoracic Surgery*.

[24] Koltuksuz U, Özen S, Uz E (1999). Caffeic acid phenethyl ester prevents intestinal reperfusion injury in rats. *Journal of Pediatric Surgery*.

[25] Winn HR, Rubio R, Berne RM (1979). Brain adenosine production in the rat during 60 seconds of ischemia. *Circulation Research*.

[26] Joannidis M, Gstraunthaler G, Pfaller W (1990). Xanthine oxidase: evidence against a causative role in renal reperfusion injury. *American Journal of Physiology*.

[27] Hassan NA, El-Bassossy HM, Mahmoud MF, Fahmy A (2014). Caffeic acid phenethyl ester, a 5-lipoxygenase enzyme inhibitor, alleviates diabetic atherosclerotic manifestations: effect on vascular reactivity and stiffness. *Chemico-Biological Interaction*.

[28] Roso NC, Correa RRM, Castiglia YMM (2012). Caffeic acid phenethyl ester effects in the kidney during ischemia and reperfusion in rats anesthetized with isoflurane. *Transplantation Proceedings*.

[29] Nath KA, Norby SM (2000). Reactive oxygen species and acute renal failure. *American Journal of Medicine*.

[30] Loef BG, Henning RH, Epema AH (2004). Effect of dexamethasone on perioperative renal function impairment during cardiac surgery with cardiopulmonary bypass. *British Journal of Anaesthesia*.

[31] Ozer MK, Parlakpinar H, Vardi N, Cigremis Y, Ucar M, Acet A (2005). Myocardial ischemia/reperfusion-induced oxidative renal damage in rats: protection by caffeic acid phenethyl ester (CAPE). *Shock*.

[32] Burdmann EA, Andoh TF, Yu L, Bennett WM (2003). Cyclosporine nephrotoxicity. *Seminars in Nephrology*.

[33] Wongmekiat O, Gomonchareonsiri S, Thamprasert K (2011). Caffeic acid phenethyl ester protects against oxidative stress-related renal dysfunction in rats treated with cyclosporin A. *Fundamental & Clinical Pharmacology*.

[34] Gökçe A, Oktar S, Yönden Z (2009). Protective effect of caffeic acid phenethyl ester on cyclosporine A-induced nephrotoxicity in rats. *Renal Failure*.

[35] Yilmaz HR, Sogut S, Ozyurt B (2005). The activities of liver adenosine deaminase, xanthine oxidase, catalase, superoxide dismutase enzymes and the levels of malondialdehyde and nitric oxide after cisplatin toxicity in rats: protective effect of caffeic acid phenethyl ester. *Toxicology and Industrial Health*.

[36] Fadillioğlu E, Erdoğan H, Söğüt S, Kuku I (2003). Protective effects of erdosteine against doxorubicin-induced cardiomyopathy in rats. *Journal of Applied Toxicology*.

[37] Saad SY, Najjar TA, Al-Rikabi AC (2001). The preventive role of deferoxamine against acute doxorubicin-induced cardiac, renal and hepatic toxicity in rats. *Pharmacological Research*.

[38] Jolivet J, Cowan KH, Curt GA, Clendeninn NJ, Chabner BA (1983). The pharmacology and clinical use of methotrexate. *The New England Journal of Medicine*.

[39] Widemann BC, Adamson PC (2006). Understanding and managing methotrexate nephrotoxicity. *Oncologist*.

[40] Aithal GP (2011). Hepatotoxicity related to antirheumatic drugs. *Nature Reviews Rheumatology*.

[41] Babiak RM, Campello AP, Carnieri EG (1998). Methotrexate: pentose cycle and oxidative stress. *Cell Biochemistry and Function*.

[42] Kintzel PE (2001). Anticancer drug-induced kidney disorders: incidence, prevention and management. *Drug Safety*.

[43] Çakir T, Özkan E, Dulundu E (2011). Caffeic acid phenethyl ester (CAPE) prevents methotrexate-induced hepatorenal oxidative injury in rats. *Journal of Pharmacy and Pharmacology*.

[44] Öktem F, Yilmaz HR, Ozguner F (2006). Methotrexate-induced renal oxidative stress in rats: the role of a novel antioxidant caffeic acid phenethyl ester. *Toxicology and Industrial Health*.

[45] Uz E, Öktem F, Yilmaz HR, Uzar E, Özgüner F (2005). The activities of purine-catabolizing enzymes and the level of nitric oxide in rat kidneys subjected to methotrexate: protective effect of caffeic acid phenethyl ester. *Molecular and Cellular Biochemistry*.

[46] Parlakpinar H, Tasdemir S, Polat A (2005). Protective role of caffeic acid phenethyl ester (CAPE) on gentamicin-induced acute renal toxicity in rats. *Toxicology*.

[47] Aygün FÖ, Akçam FZ, Kaya O, Ceyhan BM, Sütçü R (2012). Caffeic acid phenethyl ester modulates gentamicin-induced oxidative nephrotoxicity in kidney of rats. *Biological Trace Element Research*.

[48] Vardi N, Parlakpinar H, Ozturk F, Acet A (2005). Gentamicin-induced nephrotoxicity and protective effect of caffeic acid phenethyl ester in rats. *Fundamental & Clinical Pharmacology*.

[49] Levine DP (2006). Vancomycin: a history. *Clinical Infectious Diseases*.

[50] Farber BF, Moellering RC (1983). Retrospective study of the toxicity of preparations of vancomycin from 1974 to 1981. *Antimicrobial Agents and Chemotherapy*.

[51] Ingram PR, Lye DC, Tambyah PA, Goh WP, Tam VH, Fisher DA (2008). Risk factors for nephrotoxicity associated with continuous vancomycin infusion in outpatient parenteral antibiotic therapy. *Journal of Antimicrobial Chemotherapy*.

[52] Elyasi S, Khalili H, Dashti-Khavidaki S, Mohammadpour A (2012). Vancomycin-induced nephrotoxicity: mechanism, incidence, risk factors and special populations. A literature review. *European Journal of Clinical Pharmacology*.

[53] Elyasi S, Khalili H, Hatamkhani S, Dashti-Khavidaki S (2013). Prevention of vancomycin induced nephrotoxicity: a review of preclinical data. *European Journal of Clinical Pharmacology*.

[54] Ocak S, Gorur S, Hakverdi S, Celik S, Erdogan S (2007). Protective effects of caffeic acid phenethyl ester, vitamin C, vitamin E and N-acetylcysteine on vancomycin-induced nephrotoxicity in rats. *Basic and Clinical Pharmacology & Toxicology*.

[55] Öktem F, Arslan MK, Ozguner F (2005). In vivo evidences suggesting the role of oxidative stress in pathogenesis of vancomycin-induced nephrotoxicity: protection by erdosteine. *Toxicology*.

[56] Parlakpinar H, Tasdemir S, Polat A (2006). Protective effect of chelerythrine on gentamicin-induced nephrotoxicity. *Cell Biochemistry and Function*.

[57] Naidu MUR, Shifow AA, Kumar KV, Ratnakar KS (2000). Ginkgo biloba extract ameliorates gentamicin-induced nephrotoxicity in rats. *Phytomedicine*.

[58] Parlakpinar H, Özer MK, Ucar M (2006). Protective effects of caffeic acid phenethyl ester (CAPE) on amikacin-induced nephrotoxicity in rats. *Cell Biochemistry and Function*.

[59] Perry HM, Thind GS, Perry EF (1976). The biology of cadmium. *Medical Clinics of North America*.

[60] Cuypers A, Plusquin M, Remans T (2010). Cadmium stress: an oxidative challenge. *BioMetals*.

[61] Johri N, Jacquillet G, Unwin R (2010). Heavy metal poisoning: the effects of cadmium on the kidney. *BioMetals*.

[62] Cannino G, Ferruggia E, Luparello C, Rinaldi AM (2009). Cadmium and mitochondria. *Mitochondrion*.

[63] Kobroob A, Chattipakorn N, Wongmekiat O (2012). Caffeic acid phenethyl ester ameliorates cadmium-induced kidney mitochondrial injury. *Chemico-Biological Interactions*.

[64] Gong P, Chen F, Liu X, Gong X, Wang J, Ma Y (2012). Protective effect of caffeic acid phenethyl ester against cadmium-induced renal damage in mice. *The Journal of Toxicological Sciences*.

[65] Markowitz GS, Radhakrishnan J, Kambham N, Valeri AM, Hines WH, D’Agati VD (2000). Lithium nephrotoxicity: a progressive combined glomerular and tubulointerstitial nephropathy. *Journal of the American Society of Nephrology*.

[66] Gitlin M (1999). Lithium and the kidney. An updated review. *Drug Safety*.

[67] Oktem F, Ozguner F, Sulak O (2005). Lithium-induced renal toxicity in rats: protection by a novel antioxidant caffeic acid phenethyl ester. *Molecular and Cellular Biochemistry*.

[68] Bozkurt Y, Bozkurt M, Turkçu G (2012). Caffeic acid phenethyl ester protects kidneys against acetylsalicylic acid toxicity in rats. *Renal Failure*.

[69] Sundari PN, Wilfred G, Ramakrishna B (1997). Does oxidative protein damage play a role in the pathogenesis of carbon tetrachloride-induced liver injury in the rat?. *Biochimica et Biophysica Acta*.

[70] Abraham P, Wilfred G, Cathrine SP (1999). Oxidative damage to the lipids and proteins of the lungs, testis and kidney of rats during carbon tetrachloride intoxication. *Clinica Chimica Acta*.

[71] Ogeturk M, Kus I, Colakoglu N, Zararsiz I, Ilhan N, Sarsilmaz M (2005). Caffeic acid phenethyl ester protects kidneys against carbon tetrachloride toxicity in rats. *Journal of Ethnopharmacology*.

[72] Huerta C, Castellsague J, Varas-Lorenzo C, García Rodríguez LA (2005). Nonsteroidal anti-inflammatory drugs and risk of ARF in the general population. *American Journal of Kidney Diseases*.

[73] Murray MD, Brater DC, Brater DC (1993). Renal toxicity of the nonsteroidal anti-inflammatory drugs. *Annual Review of Pharmacology and Toxicology*.

[74] Rifaioglu MM, Sefil F, Gokce H, Nacar A, Dorum BA, Davarci M (2013). Protective effects of caffeic acid phenethyl ester on the dose-dependent acute nephrotoxicity with paraquat in a rat model. *Environmental Toxicology*.

[75] Baliga R, Ueda N, Walker PD, Shah SV (1999). Oxidant mechanisms in toxic acute renal failure. *Drug Metabolism Reviews*.

[76] Baliga R, Zhang Z, Baliga M, Shah SV (1996). Evidence for cytochrome P-450 as a source of catalytic iron in myoglobinuric acute renal failure. *Kidney International*.

[77] Aydogdu N, Atmaca G, Yalcin O, Batcioglu K, Kaymak K (2004). Effects of caffeic acid phenethyl ester on glycerol-induced acute renal failure in rats. *Clinical and Experimental Pharmacology and Physiology*.

[78] Orth SR (2000). Smoking—a renal risk factor. *Nephron*.

[79] Pekmez H, Ogeturk M, Ozyurt H, Sonmez MF, Colakoglu N, Kus I (2010). Ameliorative effect of caffeic acid phenethyl ester on histopathological and biochemical changes induced by cigarette smoke in rat kidney. *Toxicology and Industrial Health*.

[80] Adler S, Huang H, Wolin MS, Kaminski PM (2004). Oxidant stress leads to impaired regulation of renal cortical oxygen consumption by nitric oxide in the aging kidney. *Journal of the American Society of Nephrology*.

[81] Radke KJ (1994). Renal physiology series: part 6 of 8. The aging kidney: structure, function, and nursing practice implications. *ANNA Journal*.

[82] Eşrefoğlu M, Iraz M, Ateş B, Gül M (2012). Not only melatonin but also caffeic acid phenethyl ester protects kidneys against aging-related oxidative damage in sprague dawley rats. *Ultrastructural Pathology*.

[83] Yamashita Y, Jeschke MG, Wolf SE (2000). Differential expression of hepatocyte growth factor in liver kidney, lung, and spleen following burn in rats. *Cytokine*.

[84] Gurel A, Armutcu F, Hosnuter M, Unalacak M, Kargi E, Altinyazar C (2004). Caffeic acid phenethyl ester improves oxidative organ damage in rat model of thermal trauma. *Physiological Research*.

[85] Knave B (2001). Electromagnetic fields and health outcomes. *Annals of the Academy of Medicine Singapore*.

[86] Irmak MK, Fadillioğlu E, Güleç M, Erdoğan H, Yağmurca M, Akyol Ö (2002). Effects of electromagnetic radiation from a cellular telephone on the oxidant and antioxidant levels in rabbits. *Cell Biochemistry and Function*.

[87] Irmak MK, Oztas E, Yagmurca M, Fadillioglu E, Bakir B (2003). Effects of electromagnetic radiation from a cellular telephone on epidermal Merkel cells. *Journal of Cutaneous Pathology*.

[88] Ilhan A, Gurel A, Armutcu F (2004). Ginkgo biloba prevents mobile phone-induced oxidative stress in rat brain. *Clinica Chimica Acta*.

[89] Ozguner F, Oktem F, Armagan A (2005). Comparative analysis of the protective effects of melatonin and caffeic acid phenethyl ester (CAPE) on mobile phone-induced renal impairment in rat. *Molecular and Cellular Biochemistry*.

[90] Ozguner MF, Oktem F, Ayata A, Koyu A, Yilmaz HR (2005). A novel antioxidant agent caffeic acid phenethyl ester prevents long-term mobile phone exposure-induced renal impairment in rat. Prognostic value of malondialdehyde, N-acetyl-*β*-d-glucosaminidase and nitric oxide determination. *Molecular and Cellular Biochemistry*.

[91] Suhr D, Brummer F, Hulser DF (1991). Cavitation-generated free radicals during shock wave exposure: investigations with cell-free solutions and suspended cells. *Ultrasound in Medicine and Biology*.

[92] Morgan TR, Laudone VP, Heston WDW, Zeitz L, Fair WR (1988). Free radical production by high energy shock waves—comparison with ionizing irradiation. *Journal of Urology*.

[93] Ozguner F, Armagan A, Koyu A, Caliskan S, Koylu H (2005). A novel antioxidant agent caffeic acid phenethyl ester prevents shock wave-induced renal tubular oxidative stress. *Urological Research*.

[94] Tepel M, Aspelin P, Lameire N (2006). Contrast-induced nephropathy: a clinical and evidence-based approach. *Circulation*.

[95] Colbay M, Yuksel S, Uslan I (2010). Novel approach for the prevention of contrast nephropathy. *Experimental and Toxicologic Pathology*.

[96] Kudugunti SK, Vad NM, Ekogbo E, Moridani MY (2011). Efficacy of caffeic acid phenethyl ester (CAPE) in skin B16-F0 melanoma tumor bearing C57BL/6 mice. *Investigational New Drugs*.

[97] Chen Y-J, Liao H-F, Tsai T-H, Wang S-Y, Shiao M-S (2005). Caffeic acid phenethyl ester preferentially sensitizes CT26 colorectal adenocarcinoma to ionizing radiation without affecting bone marrow radioresponse. *International Journal of Radiation Oncology Biology Physics*.

[98] Ince H, Kandemir E, Bagci C, Gulec M, Akyol O (2006). The effect of caffeic acid phenethyl ester on short-term acute myocardial ischemia. *Medical Science Monitor*.

[99] Cagli K, Bagci C, Gulec M (2005). In vivo effects of caffeic acid phenethyl ester on myocardial ischemia-reperfusion injury and apoptotic changes in rats. *Annals of Clinical and Laboratory Science*.

[100] Iraz M, Ozerol E, Gulec M (2006). Protective effect of caffeic acid phenethyl ester (CAPE) administration on cisplatin-induced oxidative damage to liver in rat. *Cell Biochemistry and Function*.

[101] Ilhan A, Iraz M, Gurel A, Armutcu F, Akyol O (2004). Caffeic acid phenethyl ester exerts a neuroprotective effect on CNS against pentylenetetrazol-induced seizures in mice. *Neurochemical Research*.

[102] Kizilay A, Kalcioglu MT, Ozerol E (2004). Caffeic acid phenethyl ester ameliorated ototoxicity induced by cisplatin in rats. *Journal of Chemotherapy*.

[103] Ilhan A, Akyol O, Gurel A, Armutcu F, Iraz M, Oztas E (2004). Protective effects of caffeic acid phenethyl ester against experimental allergic encephalomyelitis-induced oxidative stress in rats. *Free Radical Biology and Medicine*.

[104] Özyurt H, Söğüt S, Yildirim Z (2004). Inhibitory effect of caffeic acid phenethyl ester on bleomycine-induced lung fibrosis in rats. *Clinica Chimica Acta*.

[105] Uz E, Söğüt S, Sahin S (2002). The protective role of caffeic acid phenethyl ester (CAPE) on testicular tissue after testicular torsion and detorsion. *World Journal of Urology*.

[106] Koltuksuz U, Mutus HM, Kutlu R (2001). Effects of caffeic acid phenethyl ester and epidermal growth factor on the development of caustic esophageal stricture in rats. *Journal of Pediatric Surgery*.

[107] Koltuksuz U, Irmak MK, Karaman A, Var EUA, Özyurt H, Akyol O (2000). Testicular nitric oxide levels after unilateral testicular torsion/detorsion in rats pretreated with caffeic acid phenethyl ester. *Urological Research*.

[108] Rifaioglu MM, Davarci M, Nacar A (2014). Caffeic acid phenethyl ester (CAPE) protects against acute urogenital injury following pneumoperitoneum in the rat. *Renal Failure*.

[109] Meydan S, Nacar A, Ozturk OH (2013). The protective effects of caffeic acid phenethyl ester against toluene-induced nephrotoxicity in rats. *Toxicology and Industrial Health*.

